# Serum bicarbonate concentration and the risk of death in type 2 diabetes: the Fremantle Diabetes Study Phase II

**DOI:** 10.1007/s00592-023-02130-y

**Published:** 2023-06-17

**Authors:** S. A. Paul Chubb, Wendy A. Davis, Timothy M. E. Davis

**Affiliations:** 1 PathWest Laboratory Medicine WA, Fiona Stanley Hospital, Murdoch, WA Australia; 2 Medical School, University of Western Australia, Fremantle Hospital, P. O. Box 480, Fremantle, WA 6959 Australia

**Keywords:** Serum bicarbonate, Type 2 diabetes, Mortality, Renal dysfunction, Heart rate

## Abstract

**Aims:**

To examine whether all-cause mortality is independently associated with serum bicarbonate concentration below the laboratory reference interval in a representative, well-characterised community-based cohort of people with type 2 diabetes.

**Methods:**

1478 FDS2 participants with type 2 diabetes (mean age 65.8 years, 51.6% males, median diabetes duration 9.0 years) from the longitudinal, observational Fremantle Diabetes Study Phase II (FDS2) were followed from study entry to death or end-2016. Independent associates of a low baseline serum bicarbonate (< 22 mmol/L) were determined using multiple logistic regression. The role of important covariates in influencing the association between bicarbonate and mortality was assessed by a stepwise Cox regression approach.

**Results:**

A low serum bicarbonate was associated with increased all-cause mortality in unadjusted analysis (hazard ratio (HR) 1.90 (95% confidence limits (CL) 1.39, 2.60 per mmol/L). Mortality remained significantly associated with low serum bicarbonate (HR 1.40 (95% CL 1.01, 1.94) per mmol/L) in a Cox regression model with adjustment for factors associated with mortality but not low serum bicarbonate, but inclusion of estimated glomerular filtration rate categories rendered the association non-significant (HR 1.16 (95% CL 0.83, 1.63) per mmol/L).

**Conclusions:**

A low serum bicarbonate is not an independent prognostic marker in people with type 2 diabetes but it may be a manifestation of the pathway between the development of impaired renal function and death.

## Introduction

The serum bicarbonate concentration is a commonly measured surrogate marker of acid–base status in hospitalised and community-based patients. It is well recognised that both metabolic and respiratory acid–base derangements can affect the serum bicarbonate concentration and this complicates its clinical interpretation. Nevertheless, recent studies have identified serum bicarbonate as a marker of mortality risk that is independent of other predictive variables. There is evidence that a low serum bicarbonate concentration is independently associated with all-cause mortality in representative community-based individuals [1–4] regardless of whether the cause is a metabolic acidosis or compensated respiratory alkalosis [3]. In the case of patients with chronic kidney disease (CKD) or hypertension, a low serum bicarbonate was an independent risk factor for mortality in some [5–7] but not all [8–10] studies.

Whether a low serum bicarbonate is an independent mortality risk factor among people with type 2 diabetes is uncertain. Type 2 diabetes is associated with an increased risk of CKD [11] and pulmonary dysfunction [12, 13], and relatively frequent use of diuretic and anti-hypertensive medications [14], factors that can have a complex influence on acid–base status. While most studies in the general population and in people with CKD suggest a significant link between a low serum bicarbonate and death [1–7], there is no such independent association in people with diabetic nephropathy [15], type 2 diabetes and CKD [6], or diabetes from the community [16], even if a positive association was reported in the subset of people with diabetes in another community-based cohort [1].

Apparent discrepancies between studies and across patient groups may reflect differences in selection criteria, sample sizes and thus statistical power, and/or availability of clinically important covariates which may include the existence of other, more potent, mortality risk factors in people with diabetes. The aim of the present study was, therefore, to examine whether all-cause mortality is independently associated with serum bicarbonate concentration below the reference interval in a representative, well-characterised cohort of people with type 2 diabetes from the Fremantle Diabetes Study Phase II (FDS2). We also aimed to determine whether there are covariates of serum bicarbonate that modulate its relationship with mortality and help explain inconsistencies between previous published studies.

## Materials and methods

### Participants

The Fremantle Diabetes Study Phase II (FDS2) is a prospective observational study of diabetes in a postcode-defined urban population of approximately 153,000 people living in and around the port city of Fremantle in Western Australia (WA) [17]. Of 4639 people identified as living with diabetes between 2008 and 2011, 1668 (36.0%) were recruited together with 64 participants of the Fremantle Diabetes Study Phase I who had moved out of the study area. Altogether, 1482 (85.6%) had type 2 diabetes based on demographic, anthropometric, clinical and laboratory features [18]. The present study included 1478 (99.7%) of the 1482 FDS2 participants with type 2 diabetes who had a valid measurement of the serum bicarbonate concentration at baseline.

### Clinical methods

Participants had comprehensive face-to-face assessments at baseline and biennially, interspersed with biennial postal questionnaires [17]. At each visit, demographic and clinical information was documented, physical examinations and associated investigations were carried out, and fasting blood and urine samples for biochemical tests were obtained. A body shape index (ABSI) was calculated as a more robust index of visceral obesity as a predictor of death [19]. Micro- and macrovascular complications of diabetes at study entry were identified using standard criteria [17, 20], including distal symmetric polyneuropathy (a score of > 2/8 on the clinical portion of the Michigan Neuropathy Screening Instrument [21]), retinopathy from graded fundus photographs, nephropathy (first-morning urinary albumin:creatinine ratio > 3.0 mg/mmol), renal impairment by estimated glomerular filtration rate (eGFR) determined using the Chronic Kidney Disease Epidemiology Collaboration (CKD-EPI) equation [22], coronary heart disease (CHD) (self-reported history of myocardial infarction, angina and/or revascularisation, or prior hospitalisations for these events), cerebrovascular disease (self-reported stroke/transient ischaemic attack or prior hospitalisations for these events) and peripheral arterial disease (ankle:brachial index ≤ 0.90 on either leg or diabetes-related amputation). The Charlson Comorbidity Index was calculated as a measure of chronic disease effects after excluding diabetes and diabetes-related conditions [23].

The Hospital Morbidity Data Collection (HMDC) contains information regarding all public/private hospitalisations in WA since 1970 while the Death Registrations contains information on all deaths in WA [24]. The FDS2 has been linked through the WA Data Linkage System (WADLS) to these databases, as approved by the WA Department of Health Human Research Ethics Committee, to provide validated data on incident events to end-2016.

### Biochemical assays

Morning fasting venous blood samples were collected from each patient, centrifuged promptly and analysed for standard biochemical parameters in a single nationally accredited laboratory. Spot first-morning urine samples were also collected. The homeostasis model assessment (HOMA) was used to estimate steady state beta cell function (%B) and sensitivity (%S) as percentages of a normal reference population, and insulin resistance (IR) which is the reciprocal of %S (100/%S) [25]. Serum bicarbonate was measured by the phosphoenolpyruvate carboxylase method using an Integra 800 analyser (Roche Diagnostics Australia, Castle Hill, NSW, Australia) and then an Abbott Architect ci8200 analyser (Abbott Diagnostics, Macquarie Park, NSW, Australia). Between-run imprecision (expressed as coefficient of variation) was 5.5% at 13.5 mmol/L and 3.5% at 29.6 mmol/L. The reference interval in this laboratory is 22–32 mmol/L. For the purposes of the present study, a low serum bicarbonate was defined as < 22 mmol/L. Serum potassium, creatinine, glucose, cholesterol, triglycerides and HDL-cholesterol, as well as urine albumin and creatinine, were measured by standard methods on the Integra 800 and Architect ci8200 analysers. Glycated haemoglobin was estimated by immunoassay on the Roche Integra 800 analyser throughout the study.

### Data analysis

The computer package IBM SPSS Statistics 25 (IBM Corporation, Armonk, NY, USA) was used for statistical analysis. Data are presented as percentages, mean ± SD, geometric mean (SD range), or, in the case of variables which did not conform to a normal or log-normal distribution, median and [inter-quartile range]. For independent samples, two-way comparisons for proportions were by Fisher’s exact test, for normally distributed variables by Student’s *t*-test, and for non-normally distributed variables by Mann–Whitney *U*-test. Multiple logistic regression using backward stepwise conditional modelling (*P* < 0.05 for entry, *P* ≥ 0.05 for removal), with all clinically plausible variables at *P* < 0.20 in bivariable analyses considered for entry, identified independent associates of baseline serum bicarbonate < 22 mmol/L. Cox regression was used to determine independent predictors of all-cause mortality using backward stepwise conditional modelling (*P* < 0.05 for entry, *P* ≥ 0.05 for removal) with all clinically plausible variables at *P* < 0.20 in bivariable analyses considered for entry.

To understand the relationship between a low serum bicarbonate and all-cause mortality, (1) unadjusted Cox regression was first performed with low serum bicarbonate as the exposure and all-cause mortality as the outcome (model 1); (2) adjustment was then made for age and sex (model 2); (3) further adjustment was made for those variables independently associated with all-cause mortality but not low serum bicarbonate (model 3); (4) non-significant variables in model 3 were removed using backward conditional modelling (*P* < 0.05 for entry, *P* ≥ 0.05 for removal; model 4); (5) heart rate, which was significantly associated with both low serum bicarbonate and all-cause mortality, was added to model 4 (model 5); (6) eGFR categories < 60 ml/min/1.73m^2^, which were significantly associated with both low serum bicarbonate and all-cause mortality, were added to model 4 (model 6).

## Results

### Baseline characteristics

The serum bicarbonate concentration was available for 1478 of the 1482 participants with type 2 diabetes in the FDS2 cohort (99.7%; mean age 65.8 ± 11.6 years, 51.6% males, median diabetes duration 9.0 [3.0–15.9] years). Their mean serum bicarbonate was 24 ± 2 mmol/L. The demographic, clinical and biochemical characteristics of patients with serum bicarbonate < 22 mmol/L and ≥ 22 mmol/L are summarised in Table 1. Participants with a low serum bicarbonate were more likely to be Australian Aboriginal and less likely to be Anglo-Celt, were younger at diagnosis of diabetes, had longer diabetes duration, higher fasting glucose and HbA_1c_, more intensive blood glucose-lowering treatment, greater obesity, higher heart rate, serum potassium, serum triglycerides, urinary albumin:creatinine ratio and prevalence of CHD and cerebrovascular disease, lower diastolic blood pressure, serum HDL-cholesterol and eGFR, and more comorbidities. Independent associates of a low serum bicarbonate concentration in the whole cohort are shown in Table 2. Factors that were independently associated with a low serum bicarbonate were heart rate, serum potassium, eGFR < 60 mL/min/1.73m^2^ and history of CHD, while age at diabetes diagnosis, being on insulin therapy and HDL-cholesterol were negatively associated.

**Table 1:** Baseline characteristics of participants with type 2 diabetes in the Fremantle Diabetes Study Phase II categorised by serum bicarbonate below (< 22 mmol/L) versus above (≥ 22 mmol/L) the reference range

Serum bicarbonate (mmol/L)	< 22	≥ 22	*P*-value
N (%) (total = 1478)	158 (10.7)	1320 (89.3)	
Age (years)	65.6 ± 14.4	65.8 ± 11.2	0.894
Male (%)	50.0	51.8	0.675
Ethnic background (%):			< 0.001
Anglo-Celt	41.1	54.7	
Southern European	13.9	12.5	
Other European	7.6	6.9	
Asian	2.5	4.7	
Aboriginal	15.2	6.2	
Mixed/other	19.6	15.0	
Not fluent in English (%)	10.1	10.8	0.310
Educated beyond primary school (%)	83.9	86.9	0.892
Currently married/de facto relationship (%)	60.8	62.7	0.664
Alcohol consumption (standard drinks/day)	0.1 [0–0.9]	0.1 [0–1.2]	0.140
Smoking status (%):			0.180
Never	42.9	42.4	
Ex-	42.3	47.4	
Current	14.7	10.2	
Age at diabetes diagnosis (years)	52.8 ± 13.6	55.9 ± 12.0	0.007
Diabetes duration (years)	13.6 [4.0–19.0]	8.0 [2.7–15.4]	< 0.001
Diabetes treatment (%):			0.009
Diet	15.2	25.2	
Oral medications/non-insulin injectables	60.8	52.9	
Insulin	8.9	5.2	
Insulin + oral medications/non-insulin injectables	15.2	16.8	
Fasting serum glucose (mmol/L)	7.5 [6.4–8.1]	7.1 [6.1–8.7]	0.044
HbA_1c_ (%)	7.1 [6.4–8.1]	6.8 [6.2–7.6]	0.009
HbA_1c_ (mmol/mol)	54 [46–65]	51 [44–60]	0.009
BMI (kg/m^2^)	32.0 ± 6.5	31.1 ± 6.0	0.091
Central adiposity (% by waist circumference)	78.8	70.5	0.031
ABSI (m^11/6^ kg^−2/3^)	0.082 ± 0.006	0.081 ± 0.005	0.046
Heart rate (bpm)	72 ± 14	70 ± 12	0.014
Systolic blood pressure (mm Hg)	144 ± 24	146 ± 22	0.340
Diastolic blood pressure (mm Hg)	78 ± 13	80 ± 12	0.044
Anti-hypertensive medications excluding diuretics (%)	79.5	73.0	0.084
Diuretic therapy (%)	27.6	30.6	0.462
Serum potassium (mmol/L)	4.7 ± 0.6	4.5 ± 0.5	< 0.001
Total serum cholesterol (mmol/L)	4.2 ± 1.2	4.4 ± 1.1	0.128
Serum HDL-cholesterol (mmol/L)	1.13 ± 0.29	1.24 ± 0.34	< 0.001
Serum triglycerides (mmol/L)	1.7 (1.0–2.9)	1.5 (0.9–2.5)	0.003
Lipid-modifying treatment (%)	71.8	69.0	0.521
Aspirin therapy (%)	38.7	37.5	0.793
eGFR (CKD-EPI) category (%):			< 0.001
≥ 90 ml/min/1.73m^2^	29.1	39.0	
60–89 ml/min/1.73m^2^	29.7	46.8	
45–59 ml/min/1.73m^2^	15.8	8.3	
30–44 ml/min/1.73m^2^	13.9	4.2	
< 30 ml/min/1.73m^2^	11.4	1.7	
Urinary albumin:creatinine ratio (mg/mmol)	4.8 (1.0–23.0)	3.2 (0.9–12.0)	0.003
Any retinopathy (%)	41.7	36.9	0.249
Distal symmetric polyneuropathy (%)	59.9	58.5	0.797
Peripheral arterial disease (%)	26.8	22.3	0.227
Coronary heart disease (%)	40.5	28.0	0.002
Cerebrovascular disease (%)	13.9	7.9	0.015
Charlson Comorbidity Index (%):			< 0.001
0	62.0	76.4	
1–2	22.2	16.7	
≥ 3	15.8	6.9

**Table 2:** Multiple logistic regression model of independent associates of baseline serum bicarbonate < 22 mmol/L in participants with type 2 diabetes (N = 1478). Final model, n = 1451 (98.2%)

	OR (95% CI)	*P*-value
Age at diagnosis of diabetes (increase of 10 years)	0.74 (0.63, 0.87)	< 0.001
Insulin treatment	0.57 (0.36, 0.90)	0.015
Heart rate (increase of 10 beats per minute)	1.17 (1.01, 1.34)	0.031
Serum potassium (increase of 1 mmol/L)	1.51 (1.05, 2.16)	0.025
Serum HDL-cholesterol (increase of 0.1 mmol/L)	0.92 (0.87, 0.98)	0.008
eGFR 45–59 ml/min/1.73m^2^	2.88 (1.69, 4.92)	< 0.001
eGFR 30–44 ml/min/1.73m^2^	4.84 (2.65, 8.83)	< 0.001
eGFR < 30 ml/min/1.73m^2^	7.93 (3.76, 16.74)	< 0.001
History of coronary heart disease	1.59 (1.08, 2.35)	0.018

### Serum bicarbonate and all-cause mortality

During a total of 9834 person-years (6.7 ± 1.7 years) of follow-up to end-December 2016, 272 (18.4%) of the cohort died. Baseline associates of all-cause mortality are shown in Table 3. Serum bicarbonate was negatively associated with all-cause mortality; 17.3% of those who died during follow-up had a low serum bicarbonate compared with 9.2% of survivors (*P* < 0.001). The most parsimonious Cox model of time to all-cause mortality included age, sex, marital status, ethnic background, current smoking, obesity, heart rate, estimated glomerular filtration rate categories < 60 mL/min/1.73m^2^, distal symmetric polyneuropathy, peripheral arterial disease and comorbidities (Table 4, Model 7).

**Table 3:** Baseline associates of all-cause mortality in Fremantle Diabetes Study Phase II participants with type 2 diabetes with a baseline serum bicarbonate measurement

	Alive	Deceased	*P*-value
Number	1206 (81.6)	272 (18.4)	
Age (years)	64.1 ± 10.9	73.3 ± 11.4	< 0.001
Male (%)	50.3	57.4	0.038
Education beyond primary level (%)	88.1	79.7	0.001
Not fluent in English (%)	10.0	13.6	0.102
Married/de facto (%)	65.7	48.2	< 0.001
Ethnic background (%):			
Anglo-Celt	52.5	56.6	
S. European	12.4	13.6	
Other European	7.2	5.9	0.061
Asian	5.0	2.2	
Aboriginal	6.6	9.6	
Mixed/other	16.3	12.1	
Smoking status (%):			
Never	43.7	37.2	
Ex-	46.5	48.3	0.036
Current	9.8	14.5	
Alcohol consumption (standard drinks (10 U)/day)	0.1 [0–1.2]	0.1 [0–1.5]	0.139
Age at diabetes diagnosis (years)	54.7 ± 11.7	59.6 ± 13.6	< 0.001
Diabetes duration (years)	8.0 [2.0–15.0]	14.0 [5.2–19.3]	< 0.001
Diabetes treatment (%):			
Lifestyle/diet	25.1	19.5	
OGLM^a^	54.4	51.1	0.006
Insulin alone	4.8	8.8	
Insulin + OGLM	15.7	20.6	
HbA_1c_ (%)	6.8 [6.2–7.7]	6.9 [6.2–7.8]	0.448
HbA_1c_ (mmol/mol)	51 [44–61]	52 [44–62]	0.448
Fasting serum glucose (mmol/L)	7.2 [6.2–8.9]	6.9 [5.9–8.7]	0.027
In non-insulin users (n = 1142):	(n = 951)	(n = 191)	
HOMA-2IR	1.79 (0.96–3.34)	1.54 (0.78–3.07)	0.003
HOMA-2B (%)	65.2 (35.3–120.6)	63.6 (33.6–120.5)	0.601
HOMA-2S (%)	55.7 (29.9–103.9)	64.9 (32.6–129.0)	0.003
ABSI^b^ (m^11/6^ kg^−2/3^)	0.081 ± 0.005	0.084 ± 0.006	< 0.001
Body mass index (kg/m^2^)	31.5 ± 6.0	30.1 ± 6.4	0.001
Central adiposity (% by waist circumference)	72.3	67.3	0.101
Heart rate (beats/min)	69 ± 12	74 ± 14	< 0.001
Supine systolic blood pressure (mmHg)	145 ± 21	149 ± 25	0.014
Supine diastolic blood pressure (mmHg)	80 ± 12	79 ± 14	0.043
Non-diuretic anti-hypertensive medication (%)	72.4	79.3	0.022
Diuretic use (%)	28.5	38.1	0.003
Total serum cholesterol (mmol/L)	4.4 ± 1.2	4.3 ± 1.1	0.210
Serum HDL-cholesterol (mmol/L)	1.23 ± 0.33	1.24 ± 0.39	0.794
Serum triglycerides (mmol/L)	1.5 (0.9–2.6)	1.5 (0.9–2.5)	0.294
Lipid-lowering medication (%)	68.8	71.5	0.422
Aspirin (%)	36.5	42.8	0.060
Urinary albumin:creatinine ratio (mg/mmol)	3.0 (0.8–10.8)	5.6 (1.6–20.5)	< 0.001
eGFR (mL/min/1.73m^2^):			
≥ 90	42.1	19.9	
60–89	46.1	40.1	
45–59	7.4	16.5	< 0.001
30–44	3.7	12.1	
< 30	0.7	11.4	
Any retinopathy (%)	36.1	43.2	0.039
Distal symmetric polyneuropathy (%)	54.7	76.3	< 0.001
Prior coronary heart disease (%)	25.4	47.1	< 0.001
Prior cerebrovascular disease (%)	6.7	16.5	< 0.001
Peripheral arterial disease (%)	19.9	35.6	< 0.001
Charlson Comorbidity Index (%):			
0	79.9	52.6	
1–2	14.9	27.6	< 0.001
3+	5.1	19.9	
Serum bicarbonate (mmol/L)	25 ± 2	24 ± 3	0.001
Serum bicarbonate < 22 mmol/L (%)	9.2	17.3	< 0.001

^a^Oral glucose-lowering medications and non-insulin injectables; ^b^A body shape index

**Table 4:** Cox models of time to all-cause mortality to end-2016 for participants of the Fremantle Diabetes Study Phase II with type 2 diabetes

	HR (95% CI)						
	Model 1	Model 2	Model 3	Model 4	Model 5	Model 6	Model 7
Serum bicarbonate < 22 mmol/L	1.90 (1.39, 2.60)	1.73 (1.27, 2.38)	1.38 (0.995, 1.92)	1.40 (1.01, 1.94)	1.37 (0.99, 1.90)	1.16 (0.83, 1.63)	
Age (increase of 10 years)		2.22 (1.94, 2.54)	2.06 (1.77, 2.40)	2.06 (1.77, 2.40)	2.11 (1.82, 2.45)	1.94 (1.66, 2.26)	1.98 (1.70, 2.31)
Male sex		1.36 (1.07, 1.73)	1.40 (1.07, 1.83)	1.38 (1.05, 1.81)	1.53 (1.16, 2.01)	1.40 (1.07, 1.84)	1.60 (1.21, 2.11)
Married/de facto relationship			0.68 (0.52, 0.88)	0.68 (0.52, 0.89)	0.72 (0.55, 0.93)	0.66 (0.51, 0.87)	0.69 (0.53, 0.90)
Asian ethnic background			0.59 (0.26, 1.36)				0.34 (0.15, 0.81)
Aboriginal ethnic background			3.02 (1.82, 5.00)	3.10 (1.87, 5.12)	2.76 (1.65, 4.61)	2.95 (1.80, 4.84)	2.46 (1.48, 4.09)
Current smoker			1.80 (1.23, 2.64)	1.73 (1.18, 2.54)	1.80 (1.23, 2.65)	1.82 (1.24, 2.65)	2.08 (1.41, 3.06)
ABSI* (increase of 0.001 m^11/6^/kg^2/3^)			1.04 (1.01, 1.06)	1.04 (1.01, 1.06)	1.03 (1.01, 1.06)	1.03 (1.001, 1.05)	1.03 (1.00, 1.05)
Distal symmetric polyneuropathy			1.39 (1.04, 1.87)	1.40 (1.05, 1.88)	1.40 (1.05, 1.89)	1.39 (1.03, 1.86)	1.37 (1.02, 1.85)
Peripheral arterial disease			1.31 (1.01, 1.70)	1.33 (1.02, 1.72)	1.38 (1.06, 1.79)	1.34 (1.04, 1.75)	1.37 (1.06, 1.79)
Charlson Comorbidity Index 1 or 2			1.74 (1.30, 2.33)	1.76 (1.32, 2.35)	1.73 (1.30, 2.31)	1.59 (1.19, 2.14)	1.49 (1.11, 2.01)
Charlson Comorbidity Index ≥ 3			2.75 (1.97, 3.84)	2.71 (1.94, 3.78)	2.39 (1.71, 3.35)	2.00 (1.38, 2.89)	1.76 (1.22, 2.55)
Heart rate (increase of 10 beats/min)					1.27 (1.16, 1.39)		1.29 (1.18, 1.41)
eGFR 45–59 mL/min/1.73m^2^						1.63 (1.15, 2.31)	1.74 (1.23, 2.47)
eGFR 30–44 mL/min/1.73m^2^						1.62 (1.08, 2.44)	1.59 (1.06, 2.36)
eGFR < 30 mL/min/1.73m^2^						3.21 (2.01, 5.13)	4.24 (2.64, 6.82)

Model 1, Serum bicarbonate <22 mmol/L; Model 2, Model 1 plus age and sex; Model 3, Model 1 plus variables in the most parsimonious model (Model 7) that are not also associated with serum bicarbonate <22 mmol/L; Model 4, Model 3 but with backward conditional modelling to remove non-significant variables; Model 5, Model 4 plus heart rate; Model 6, Model 4 plus eGFR categories <60 ml/min/1.73m^2^; Model 7, Most parsimonious model

*ABSI, A body shape index

In unadjusted Cox regression, participants with a low serum bicarbonate were nearly twice as likely to die (HR (95% CI): 1.90 (1.39, 2.60), *P* < 0.001; Table 4, Model 1). In multivariable Cox regression models, the association of low serum bicarbonate with mortality remained significant after adjustment for age and sex (1.73 (1.27, 2.38), *P* < 0.001; Table 4, Model 2). When variables independently associated with mortality but not with low serum bicarbonate were entered and non-significant variables removed, the association of a low serum bicarbonate with mortality was attenuated but remained statistically significant (1.40 (1.01, 1.94), *P* = 0.044; Table 4, Model 4). Further separate adjustment for heart rate and low eGFR categories rendered the association of a low serum bicarbonate with mortality non-significant (*P* = 0.058 and 0.397, respectively; Table 4, Models 5 and 6), although the change in risk when heart rate was added was small.

## Discussion

The present study shows that a baseline serum bicarbonate concentration < 22 mmol/L in community-based people with type 2 diabetes was a significant predictor of subsequent all-cause mortality in statistical models incorporating limited adjustment for confounding variables. However, this association was non-significant in fully adjusted models that included impaired renal function. These observations suggest that a low serum bicarbonate may a manifestation of the causal pathway between renal impairment and death, but that it is not an independent risk factor for mortality in type 2 diabetes.

There appears to be a distinction between the consistent finding that a low serum bicarbonate is associated with the risk of death in representative general population samples after adjustment for relevant covariates including eGFR [1–4] and the inconsistent but largely negative results in studies of people with CKD [5–8, 10] or diabetes [1, 6, 16]. In the present study, we developed models which adjusted for increasing numbers of clinically relevant covariates and, in accord with post hoc analyses of two large two angiotensin II receptor blocking agent trials in people with diabetic nephropathy [15], found that eGFR abrogated the relationship between serum bicarbonate and death.

A possible explanation for these different observations is that, in people from the general population who do not have CKD or diabetes, a chronic low grade metabolic acidosis, for which a low serum bicarbonate is a surrogate, has pathophysiological consequences including accelerated renal damage [26], protein catabolism [27], systemic inflammation [28] and activation of the renin-angiotensin system [29]. These effects are amplified and swamped by the dominant adverse metabolic consequences of CKD, which is also associated with exacerbation of major conventional cardiovascular disease risk factors including hypertension and dyslipidaemia [30]. This is likely to be the case in diabetes as well, although there is some evidence that that a low serum bicarbonate in an individual with diabetes is not as strongly associated with kidney disease progression and mortality as in people with CKD but without diabetes [31]. Nevertheless, there was an independent graded positive association between baseline eGFR and a low serum bicarbonate in our participants even if the cross-sectional nature of the analysis does not allow distinguishing cause from consequence.

There were other significant independent associates of a serum bicarbonate < 22 mmol/L in the present study. The association with a younger age at diagnosis likely reflects the effect of accelerated cellular ageing in diabetes [32] which could exacerbate the age-related increase in the serum bicarbonate [33]. The inverse association between insulin therapy and a low serum bicarbonate can be explained by its known effect of inhibiting adipocyte lipolysis and thereby reducing serum free fatty acid and ketoacid concentrations [34]. Evidence from in vitro to in vivo studies suggests that acidosis may cause decreased cardiomyocyte contractility and a reflex increased heart rate [35–37], consistent with the positive association between low serum bicarbonate and heart rate in the present and previous [1, 16] studies. An increase in heart rate is also a known risk factor for cardiovascular disease and death in general populations studies, as demonstrated in a recent meta-analysis showing an increase in risk for all-cause mortality of 17% per 10 beats/minute increase [38] compared with a similar 29% per 10 beats/minute increased risk among our participants. Interestingly, no other studies of the association of serum bicarbonate with mortality have included heart rate as a covariate. An association between serum potassium and a low serum bicarbonate is well recognised, including in older people with renal impairment [39], as is an inverse association between serum HDL-cholesterol and a low serum bicarbonate [9] and between CHD and a low serum bicarbonate [16].

Our study had some limitations. We used the serum bicarbonate as a surrogate measure of metabolic acidosis even though respiratory alkalosis is a possible cause of low bicarbonate. In common with most relevant studies to date, we did not have measures of blood pH or pCO_2_ so cannot make the distinction between these states but our results with respect to associations of a low serum bicarbonate with factor such as renal impairment, hyperkalaemia and increased heart rate are consistent with the published evidence. We did not have enough incident cases of CHD or incident heart failure to make meaningful analyses of the relationship between bicarbonate and these outcomes. On the other hand, the present study is one of few of the association of bicarbonate and mortality to be carried out in a representative community-based cohort of people with type 2 diabetes, with extensive clinical and biochemical characterisation of the participants. Model adjustment utilised the most parsimonious approach, thus reducing the risk of over-adjustment.

The clinical implications of our findings are that a low serum bicarbonate, measured as part of routine care, is a crude mortality risk factor that may indicate need for intensified cardiometabolic management in people with type 2 diabetes. In most, similar information will be obtained from the eGFR as calculated from serum creatinine measurement. However, in some groups of people, particularly frail elderly patients and those who have suffered significant limb amputations in whom eGFR may be overestimated by serum creatinine, a serum bicarbonate may be complementary or even better marker of mortality risk.

In conclusion, in our community-based cohort of people with type 2 diabetes, a serum bicarbonate below the laboratory reference interval was bivariately significantly associated with all-cause mortality, but adjustment for important confounders including renal dysfunction abrogated this association. Our results are consistent with serum bicarbonate being one manifestation of the pathway from impaired renal function to death in people with type 2 diabetes. A low serum bicarbonate in a patient being cared for in the community may be a valid marker of increased risk of death, especially in patients with complications of advanced diabetes such as sarcopenia or amputation in whom the calculated eGFR may be less reliable.

## Data Availability

Restrictions apply to the availability of data generated or analysed during this study to preserve patient confidentiality or because they were used under license. The corresponding author will on request detail the restrictions and any conditions under which access to some data may be provided.
